# Reducing Brain Injury of Preterm Infants in the Delivery Room

**DOI:** 10.3389/fped.2018.00290

**Published:** 2018-10-16

**Authors:** Francesca Viaroli, Po-Yin Cheung, Megan O'Reilly, Graeme R. Polglase, Gerhard Pichler, Georg M. Schmölzer

**Affiliations:** ^1^Centre for the Studies of Asphyxia and Resuscitation, Royal Alexandra Hospital, Edmonton, AB, Canada; ^2^Department of Pediatrics, University of Alberta, Edmonton, AB, Canada; ^3^The Ritchie Centre, Hudson Institute of Medical Research and Department of Obstetrics and Gynaecology, Monash University, Melbourne, VIC, Australia; ^4^Department of Pediatrics, Medical University Graz, Graz, Austria

**Keywords:** infants, newborn, neonatal resuscitation, very low birth weight infants, brain injury, intraventricular hemorrhage

## Abstract

Cerebrovascular injury is one of the major detrimental consequences of preterm birth. Recent studies have focused their attention on factors that contribute to the development of brain lesions immediately after birth. Among those factors, hypothermia and lower cerebral oxygen saturation during delivery room resuscitation and high tidal volumes delivered during respiratory support are associated with increased risk of severe neurologic injury. In preterm infants, knowledge about causes and prevention of brain injury must be applied before and at birth. Preventive and therapeutic approaches, including correct timing of cord clamping, monitoring of physiological changes during delivery room resuscitation using pulse oximetry, respiratory function monitoring, near infrared spectroscopy, and alpha EEG, may minimize brain injury, Furthermore, postnatal administration of caffeine or other potential novel treatments (e.g., proangiogenic therapies, antioxidants, hormones, or stem cells) might improve long-term neurodevelopmental outcomes in preterm infants.

## Introduction

Cerebrovascular lesions including hemorrhagic and ischemic injuries are major detrimental consequences of preterm birth resulting in adverse neurodevelopmental outcomes. There is increasing evidence that fluctuations in cerebral blood flow and regional cerebral oxygenation, delivery of respiratory support, and changes in temperature in the delivery room (DR) initiates an inflammatory cascade leading to brain injury (1–3). The premature brain is vulnerable to oxidative stress from hypoxic–ischemic injury, which depends on heart-rate/cardiac output, immature vascular supply, and impairment in vascular autoregulation (4). In this review, we describe what is known about the factors involved in the development of brain injury during fetal-to-neonatal transition at birth. Furthermore, we discuss potential preventive and therapeutic approaches, which might help to minimize brain injury during neonatal resuscitation.

## Temperature

The World Health Organization (WHO) defines hypothermia in newborn infants as (i) cold stress or mild hypothermia between 36.0 and 36.4°C (96.8–97.5°F), (ii) moderate hypothermia between 32.0 and 35.9°C (89.6–96.6°F), and (iii) severe hypothermia <32°C (89.6°F) (5). The WHO recommend that the temperature of healthy newborns should be maintained between 36.7 and 37.7°C, as a decrease in body temperature by one degree will increase mortality by ~28% (5). Moderate hypothermia can further result in lower partial pressure of arterial oxygen, metabolic acidosis, and an increased risk of intraventricular hemorrhage (IVH) (6, 7).

Preterm infants are at risk of hypothermia because of their large surface area to body mass ratio, thin and immature skin, and a lack of brown adipose tissue. Standard delivery room practices to prevent heat loss include (i) set ambient temperature, (ii) polyethylene occlusive wraps, and (iii) exothermic mattresses for preterm infants less than 32 weeks' gestation (8). A recent study by Garcia-Munoz Rodrigo et al. (6) reported increased mortality and rates of IVH (≥ grade III) in 635 infants born less than 30 weeks' gestation with hypothermia at Neonatal Intensive Care Unit (NICU) admission. This is concerning as hypothermia rates remain high in this population, with 40–50% of extreme low birth weight (ELBW) infants presenting with a core temperature below 36.0°C upon admission to the NICU (9, 10).

A relatively overlooked aspect of heat loss in the DR is the use of unconditioned gases during respiratory support. Unconditioned gases are “cold and dry,” typically room temperature (23°C) with very low relative humidity (2–5%; ambient relative humidity is usually 30–40%) (11). This differs from standard practice in the NICU where medical gases are heated and humidified (conditioned) during respiratory support (11). A cohort study by te Pas et al. compared 112 infants <32 weeks' gestation before (the “cold” cohort) and after (the “heated” cohort) the introduction of heated and humidified gases during respiratory support at birth (12). The study reported significantly improved NICU admission temperature following the use of heated and humidified gases in the DR. Furthermore, a trial by the same group in 203 infants <32 weeks' gestation reported an ~50% reduction in rates of major IVH (grades III and IV) 5 vs. 9.5% in infants randomized to conditioned gases compared to unconditioned gases, respectively (13). Similarly, McGrory et al. randomized infants born 30 weeks' gestation to conditioned or unconditioned gases and reported a 66% reduction in IVH in the conditioned group (11). These data suggest that respiratory support in the DR should be provided using conditioned gases to reduce risk of brain injury.

## Oxygen saturation

Immediately after birth arterial oxygen saturation (SpO_2_) values in the newborn could be as low as 30% (14), which then increase over the next 7–10 min to 85–95% (15). In 2010, the resuscitation guidelines were revised to initiate the resuscitation of term infants using air, and either air or <65% oxygen for preterm infants (16). Subsequently oxygen concentrations should be adjusted to meet SpO_2_ ranges, which are similar to those of spontaneously breathing, healthy full-term infants. However, the change to using lower oxygen concentration is based on limited evidence. Indeed, in a *post hoc* analysis the TO2RPIDO trial (17) reported that infants <28 weeks' gestation receiving initially air had an almost four-fold increased risk of death [RA: 10 of 46 [22%]] compared with those given 100% oxygen. Similarly, Rook et al. compared 30 vs. 65% oxygen and reported a significantly faster time to reach an SpO_2_ of 88% [median (IQR) 3:14 (2:08–5:24) vs. 5:45 (3:49–7:51) min] (18). This is important as a recent individual patient analysis of eight trials demonstrated that 46% of preterm infants resuscitated with an initial low oxygen concentration did not reach SpO_2_ of 80% at 5 min, which was associated with increased risk of major IVH (19). Of interest, while it took a longer time to achieve the target SpO_2_ when low oxygen concentration was initiated in the resuscitation, there was an almost five times higher risk of death when there was bradycardia at 5 min of life. Furthermore, a review of a Canadian population of infants <29 weeks' gestation reported a higher risk of severe neurologic injury or death when resuscitation was started with low oxygen (20). Similarly, a recent survey of 630 clinicians from 25 countries reported that the majority would initiate preterm infant DR stabilization using 30–40% oxygen (16). These data suggest that if lower starting oxygen concentrations are used, a potentially more aggressive oxygen titration would be required to avoid hypoxia (as well as hyperoxia) that is associated with IVH or brain injury. Initial low oxygen concentration during resuscitation might not be optimal and large trials are urgently needed to address this knowledge gap. Most recently PRESOX (NCT01773746) comparing 21 vs. 60% oxygen at birth has been halted due to lack of recruitment. The STARTPreterm (NCT03115463) and TORPIDO2 ACTRN12615000115538) trials are currently ongoing to address optimized targeted oxygen strategies during DR resuscitation and their effects on developmental outcome.

## Cerebral tissue oxygenation

Arterial SpO_2_ and heart rate are routinely used to assess oxygen delivery and oxygen consumption for preterm infants. However, both parameters only indirectly assess cerebral oxygen demand. Tissue oxygenation is influenced by oxygen delivery and oxygen consumption, where oxygen delivery could be affected by changes in SpO_2_, arterial hemoglobin content, and cerebral perfusion. Cerebral perfusion itself relies on adequate cardiac output and regional vascular resistance, where cerebral vascular resistance is affected by autoregulatory capacities. Routine SpO_2_ monitoring alone only assesses the oxygen saturation of the arterial blood which reaches the brain, therefore it does not reflect oxygen delivery, which is also dependent on perfusion and hemoglobin content. Near-Infrared spectroscopy (NIRS) assesses the difference in absorption of near-infrared light by oxy- and deoxyhemoglobin to measure the oxygen saturation in regional tissue. This allows for a continuous non-invasive measurement of regional cerebral oxygen saturation (crSO2) and cerebral oxygen extraction (FTOE) (21). Reference ranges of rScO2 and FTOE in term and preterm infants requiring no medical support during transition immediately after birth have been established (22): in all neonates, median crSO2 was 41% (23–64) at 2 min, 68% (45–85) at 5 min, 79% (65–90) at 10 min, and 77% (63–89) at 15 min of age. In Pichler's study, infants with IVH had significantly lower cerebral oxygenation compared to infants without IVH, whereas SpO_2_ was similar especially after 10 min in both groups. This is further supported by a recent randomized trial comparing oxygen titration using NIRS and SpO_2_ vs. SpO_2_ alone (23). Overall, the burden of cerebral hypoxia in was halved when titration was done based on NIRS and SpO_2_, and a trend to lower mortality and cerebral injury in NIRS and SpO_2_ group was observed (23). Furthermore, the SafeboosC study compared cerebral NIRS monitoring in combination with a dedicated treatment guideline during the first 72 h of life to blinded NIRS oxygenation monitoring with standard care in infants <28 weeks' gestation (24). The 86 infants randomized to the NIRS group had a 58% (95% confidence interval 35–73%, *P* < 0.001) reduction in their burden of hypoxia and hyperoxia compared with the control group, but no difference in their long-term-outcome. Taken together, cerebral oxygenation could be optimized using a dedicated treatment guideline in combination with cerebral NIRS monitoring (24). A recent two-center observational cohort study reported that infants <32 weeks' gestation with low crSO_2_-values had significantly higher rates of IVH compared to infants with normal crSO_2_ values (25). Furthermore, there is increasing evidence that cerebral tissue oxygenation is significantly lower when resuscitation interventions are needed compared to unassisted transition (26). In addition, cerebral FTOE values were significantly elevated in infants receiving respiratory support, suggesting compensation for a lower cerebral oxygen delivery (27). These data suggest that additional monitoring using crSO_2_ during the immediate transition period might reduce the risk of developing IVH. Monitoring oxygen delivery using arterial pulse oximetry alone might be insufficient to identify preterm infants with cerebral hypoxia and at risk for IVH. Adding NIRS monitoring might reduce cerebral hypoxia and the incidence of premature infants developing IVH. A large trial is currently underway (COSGOD III-NCT03166722) to address this knowledge gap (28).

## Tidal volume

Most preterm infants born <28 weeks' gestation require some form of respiratory support in the DR (29), which is commonly delivered using positive pressure ventilation (PPV). During PPV a fixed pressure is chosen with the assumption it will deliver an adequate tidal volume (V_T_), but the V_T_ is rarely measured (8). There is ample evidence that high V_T_ delivery during PPV causes ventilator-induced lung injury (30, 31). In addition, several animal studies reported that the delivery of V_T_ >8 mL/kg causes *ventilation induced brain injuries (VIBI)* in preterm infants (2, 3, 32). Polglase et al. (32) reported that a high V_T_ strategy (V_T_ of >8 mL/kg) compared to a low V_T_ strategy (V_T_ of 5–7 mL/kg) during initial resuscitation in preterm lambs causes increased cerebral hemodynamic instability, increased brain inflammation, oxidative stress, and vascular extravasation. These changes are related to up-regulation of systemic pro-inflammatory cytokines (e.g., IL-1b, IL-6, and TNF-a), which compromises the integrity of the blood-brain-barrier and neurovascular circulation (2, 3, 32). In a recent observational study in 165 preterm infants <29 weeks' gestation, Mian et al. (1) observed a four-fold increase in IVH in preterm infants ventilated with V_T_ > 6 mL/kg^1^. Indeed, 75% of included infants had high V_T_ and 41 (25%) normal V_T_. Overall, severe IVH (grade III or IV) developed in 33/124 (27%) infants in the high V_T_ group and 2/41 (6%) in the normal V_T_ group (*P* = 0.01). These results are supported by animal studies in preterm lambs who reported an increase in brain inflammation, oxidative stress, vascular extravasation, and brain inflammation when using V_T_ > 8 mL/kg during initial resuscitation. Despite the causative relationship between V_T_ and IVH remains to be determined, it is prudent to investigate if using direct feedback systems (e.g., a respiratory function monitor) would help minimize VIBI in preterm infants.

## Implementing available knowledge

Implementation of knowledge regarding the causes of brain injury in the preterm infant should be initiated at birth, rather than later when admitted into the NICU, as this may be the key to prevention.

### Placental transfusion and cord management

The optimal timing for clamping the umbilical cord after birth in preterm infants remains unclear. For centuries, delayed cord clamping (DCC) was practiced until a change in the 1950's to immediate cord clamping (ICC), which allowed a rapid transfer of the newborn to neonatal clinicians. However, ICC is associated with significantly lower preload and afterload, higher pulmonary vascular resistance, persistent right-to-left atrial and ductal shunting, and fluctuations in cerebral blood pressure and cerebral blood flow, which might cause cerebral ischemia or hemorrhage (33). This is supported by several animal studies demonstrating that DCC results in improved cardiovascular and cerebral stability (33–35). Furthermore, randomized trials and meta-analyses reported a trend of reducing major IVH, in addition to a significant reduction in mortality, with DCC compared to ICC (36, 37). Alternatively, umbilical cord milking (UCM), which allows for rapid transfer of blood from the placenta to the infant (38), has been proposed. A recent animal study raised concerns about the hemodynamic and cerebral effects of UCM and reported that UCM without placental refill failed to provide placental transfusion, and UCM strategies caused considerable hemodynamic disturbances in carotid artery blood flow and systemic blood pressure without increasing pulmonary blood flow (33). The study concluded that UCM does not provide the same physiological benefits as physiological-based cord clamping. However, a recent trial comparing UCM with DCC reported higher hemoglobin, first infants' temperature in the DR, and systemic blood flow with UCM in preterm infants (39). Furthermore, infants randomized to UCM had higher language and cognitive scores compared to DCC, and similar rates of mild or moderate to severe neurodevelopmental impairment (40). These data are very promising and the currently ongoing larger trials including PREMOD2 (NCT03019367) in 23^+0^ to 31^+6^ and MINVI (NCT03631940) in 35^+0^ to 41^+6^ weeks' gestation will provide a more definite answer.

More recently, animal data suggest that initiating ventilation prior to umbilical cord clamping increases pulmonary blood flow, and subsequently left ventricular output, and improves arterial and cerebral oxygenation, therefore leading to a smoother cardiovascular transition (34, 35, 41). Initiating ventilation prior to umbilical cord clamping in preterm infants is feasible and safe (42–44). In addition, none of the initiating ventilation prior to umbilical cord clamping human clinical trials have reported any beneficial outcomes. Therefore, larger studies adequately powered to examine important short- and long-term outcomes are needed (45). The currently ongoing study (Vent FIRST-NCT02742454) will examine some of these outcomes.

### Monitoring

#### Respiratory function monitoring

During mask ventilation in the DR, large and variable V_T_s are often delivered (46–54). Schmölzer et al. reported a median (IQR) V_T_ of 8.7 ml/kg (5.3–11.3) which varied widely during each resuscitation and between resuscitators (46, 49, 50). Similarly, Kaufman et al reported median V_T_s of 8.3–9.3 mL/kg during PPV (47). These V_T_s were significantly larger compared to V_T_s during spontaneous breathing (52, 55, 56). Furthermore, van Vonderen et al (54) reported a median (IQR) V_T_ of 8.3 (6.8–15.4) during mask PPV in preterm infants <32 weeks' gestation in the DR. Using a respiratory function monitor (RFM) would allow for an objective assessment of mask PPV (48, 50, 51, 53). Despite the technicality in its application, a RFM would provide real-time information of mask leak, airway obstruction, and V_T_ delivery which are important in neonatal resuscitation and ventilatory support (48). A small randomized trial comparing the display of a RFM visible or masked reported lower rate of excessive V_T_ in the infants with the RFM visible (57). However, the trial included all infants <32 weeks' and therefore did not report any difference in brain injury. Trials examining if an RFM would decrease rates of IVH during PPV in the DR are urgently needed.

#### Closed-loop automatic oxygen control

Premature infants often require oxygen supplementation immediately after birth to achieve oxygen saturation targets and to avoid hypoxemia. However, the titration of oxygen often leads to hyperoxemia, which causes systemic and tissue oxidative stress and can impair long-term outcomes (58–61). Preterm infants are monitored using pulse oximetry to adjust the fraction of inspired oxygen (FiO_2_) to reach the targeted oxygen saturation. Recently, closed loop FiO_2_ systems have been utilized, which consist of an oxygenation monitoring device or pulse oximeter, a gas delivery device, and an algorithm that determines the timing, degree, and frequency of each FiO_2_ adjustment to keep SpO_2_ within target range set by the clinician. The closed loop FiO_2_ control systems minimize exposure to both high or low O_2_ levels and avoid large fluctuations in SpO_2_ levels. The CLAC trial (62) randomized 34 preterm infants <37 weeks' gestation to routine manual control (RMC) or RMC supported by CLAC using a cross-over design for 24-h periods. Overall, the median time with arterial oxygen saturation levels within target range was 61.4% for RMC and 71.2% for CLAC (*p* < 0.001). Also, the median (range) number of manual FiO_2_ adjustments was significantly reduced for RMC compared to CLAC (77 vs. 52%, respectively; *p* = 0.007). A recent randomized cross-over trial in 41 preterm infants evaluated time spent within a predefined SpO_2_ alarm range using three different SpO_2_ target ranges (86–94% vs. 88–92% vs. 89–91%) during non-invasive respiratory support (63). Overall, the duration within the SpO_2_ target range and hyperoxemia was similar. However, duration of severe hypoxemia (SpO_2_ < 80%) was significantly reduced when 88–92% or 89–91% was used as a target range compared to 86–94%, with 1.9, 1.7, and 3.4% (*p* < 0.001), respectively. This study suggests that a narrowing of the target range might reduce duration of hypoxemia, without increasing risk of hyperoxemia. However, no study has been performed in the DR and its role in optimizing systemic and tissue oxygenation remains unclear.

#### Alpha-EEG

Alpha-EEG (aEEG) provides a non-invasive continuous assessment of cerebral activity and recently there has been an increasing interest in monitoring cerebral activity during the fetal-to-neonatal transition and its' relationship with cerebral injury and long-term neurodevelopmental outcomes. Pichler et al showed that during immediate transition in infants >34 weeks' gestation, infants requiring resuscitation at birth showed a depressed cerebral activity along with cerebral oxygenation. Similarly, Tamussino et al. (64) reported that term infants with lower cerebral activity had lower cerebral oxygenation with increased cerebral oxygen extraction when compared to infants with normal aEEG values within the first 15 min after birth. As oxygen delivery and oxygen extraction is related to subsequent brain damage and long-term neurodevelopment impairment, cerebral monitoring combining aEEG and NIRS might allow for early identification of infants at risk of developing brain damage.

### Drugs

#### Caffeine

Caffeine is routinely used to prevent apnea of prematurity, and it has been shown to improve neurodevelopmental impairment (65–67). The caffeine for apnea of prematurity trial by Schmidt et al. (67) reported reduced incidence of cerebral palsy (4.4 vs. 7.3%; adjusted odds ratio, 0.58; 95% CI, 0.39–0.87; *p* = 0.009) and cognitive delay (33.8 vs. 38.3%; adjusted odds ratio, 0.81; 95% CI, 0.66–0.99; *p* = 0.04) in infants treated with caffeine compared to placebo (68).

In a recent pilot study by Katheria et al. (69) the administration of caffeine to 21 preterm neonates <29 weeks' gestation within 2 h after birth improved blood pressure (*p* = 0.03) and systemic blood flow (superior vena cava flow, *p* = 0.04 and right ventricular output, *p* = 0.03) when compared to late administration (at 12 h of age), and there was no difference in the need for intubation (*p* = 0.08) or vasopressors (*p* = 0.21) by 12 h of age. Furthermore, Dekker et al. (70) showed that among preterm neonates <29 weeks' gestation a loading dose of caffeine in the DR significantly increased minute volume at 7–9 min after birth (*p* < 0.05) when compared to caffeine administration later in the NICU. Furthermore, there may be a neuroprotective effect of caffeine in the developing brain which is related to anti-inflammatory action, oxidative stress modulation (65, 66). Further studies in the early administration of caffeine regarding the benefits and impact on the developing brain are needed.

#### Proangiogenic therapies, antioxidants, hormones, and stem cells

Proangiogenic therapies (e.g., vascular endothelial growth factor or fibroblast growth factor) (71), antioxidants (e.g., allopurinol, erythropoietin) (72–75), hormones (e.g., melatonin) (76), and stem cells or stem cell factors (77) have all been described as potential therapeutic strategies to promote functional recovery after brain injury. While these therapies seem promising, their application to reduce VIBI during the initial resuscitation in the DR is questionable and clinical trials are needed to confirm their feasibility, safety, and effectiveness.

## Discussion

There is increasing evidence that suboptimal management during the fetal to neonatal transition contributes to the development of preterm brain injury. Factors associated with preterm brain injury include hypothermia, hyperoxia with increased oxygen load and increased oxygen radicals, high V_T_ delivery during DR resuscitation, and hypoxia with low cerebral tissue oxygenation. Improved DR care has the potential to improve short- and long-term outcomes of preterm infants.

It is important to ensure that the ambient temperature of DR is appropriate and infant's bed is properly prepared. Upon delivery of the preterm infant, DCC should be practiced. Studies are needed to optimize cord management by examining the effects of UCM or DCC on cerebral blood flow and hemodynamics or physiological based cord clamping (e.g., ventilation prior to cord clamping), which might improve cardiovascular transition and therefore reduce brain injury. The clinical team must carefully monitor the infant's temperature and employ strategies (e.g., polyethylene bag, heating mattress) to reduce the risks of hypo- and hyperthermia. Supplemental oxygen should be given judiciously and guided by pulse oximetry and current oxygen targets to reduce exposure to hypoxia and hyperoxia. Similarly, adding NIRS might allow the clinical team to identify infants at risk of developing IVH through assessing cerebral tissue hypoxia/hyperoxia (78). When preterm infants fail to breathe adequately immediately after birth, adequate mask PPV will create a functional residual capacity and facilitate gas exchange. However, in order to reduce the risks of VIBI, the clinical team should avoid high V_T_ delivery, which could be assisted by using a RFM.

At this time, the evidence is preliminary and long-term neurodevelopmental outcomes are lacking. This does not support a recommendation for monitoring of cerebral activity during the immediate transition after birth nor the postnatal administration of therapeutic drugs including caffeine and other potential agents.

## Author contributions

GS and FV: conception and design; FV, GS, P-YC, MO'R, GRP and GP: collection and assembly of data, analysis and interpretation of the data, drafting of the article, critical revision of the article for important intellectual content, and final approval of the article.

### Conflict of interest statement

The authors declare that the research was conducted in the absence of any commercial or financial relationships that could be construed as a potential conflict of interest.

## Abbreviations

SpO_2_: Oxygen saturation
IVH: Intraventricular hemorrhage
NICU: Neonatal Intensive Care Unit
ELBW: Extreme low birth weight
PPV: Positive pressure ventilation
DR: Delivery room
V_T_: Tidal volume
aEEG: Alpha-EEG
NIRS: Near-Infrared spectroscopy
DCC: Delayed cord clamping
ICC: Immediate cord clamping
UCM: Umbilical cord milking
rScO2: Cerebral oxygen saturation
FTOE: Cerebral oxygen extraction
MgSO_4_: Magnesium sulfate.
